# Oral Health Knowledge and Training of French Community Pharmacy Students

**DOI:** 10.3290/j.ohpd.c_2813

**Published:** 2026-09-09

**Authors:** Lise Gentet, Lucile Barré, Alexia Marie-Cousin, Sylvie Jeanne, Sarah Dion, Solen Novello

**Affiliations:** a Lise Gentet Doctor of Dental Surgery, Unité de Formation et de Recherche d’Odontologie, Université de Rennes – Campus Villejean, 2 Av. du Professeur Léon Bernard, 35043 Rennes. Conceptualisation, methodology, validation, formal analysis, investigation, writing – original draft, visualisation.; b Lucile Barré Doctor of Dental Surgery, Unité de Formation et de Recherche d’Odontologie, Université de Rennes – Campus Villejean, 2 Av. du Professeur Léon Bernard, 35043 Rennes. Conceptualisation, methodology, validation, formal analysis, investigation, writing – original draft, visualisation.; c Alexia Marie-Cousin Associate Professor, Unité de Formation et de Recherche d’Odontologie, Université de Rennes – Campus Villejean, 2 Av. du Professeur Léon Bernard, 35043 Rennes; Pôle d’Odontologie, CHU Rennes, 35000 Rennes, France. Conceptualisation, writing – review and editing; d Sylvie Jeanne Professor, PhD, Unité de Formation et de Recherche d’Odontologie, Université de Rennes – Campus Villejean, 2 Av. du Professeur Léon Bernard, 35043 Rennes; Pôle d’Odontologie, CHU Rennes, 35000 Rennes, France; ISCR (Institut des Sciences Chimiques de Rennes) UMR 6226, Univ Rennes, Rennes, France. Conceptualisation, writing – review and editing; e Sarah Dion Associate Professor, PhD, Inserm, EHESP, Irset (Institut de recherche en santé, environnement et travail) – UMR _S 1085 – Université de Rennes, Rennes, France. Conceptualisation, writing – review and editing; f Solen Novello Associate Professor, PhD, Unité de Formation et de Recherche d’Odontologie, Université de Rennes – Campus Villejean, 2 Av. du Professeur Léon Bernard, 35043 Rennes, France; Pôle d’Odontologie, CHU Rennes, 35000 Rennes, France; ISCR (Institut des Sciences Chimiques de Rennes) UMR 6226, Univ Rennes, Rennes, France. Conceptualisation, methodology, validation, formal analysis, writing – review and editing, visualisation, supervision.

**Keywords:** dentistry, initial training, interprofessionalism, oral health, pharmacy

## Abstract

**Purpose:**

To investigate the sixth-year community pharmacy French students’ perception of their oral health education and knowledge.

**Methods and Materials:**

An analytical cross-sectional study was conducted using a questionnaire sent by e-mail to sixth-year community pharmacy students of 20 faculties of pharmacy. Data were collected online, and descriptive data analyses were conducted.

**Results:**

A total of 318 students responded to the survey. The majority (n = 308) felt that courses in dentistry would be useful for their future practice. Two-thirds of students (n = 228) rated their perceived knowledge as ‘insufficient’ or ‘non-existent’. More than half reported feeling uncomfortable when facing a patient presenting to the pharmacy with an oral health complaint or pathology. Overall, participants emphasised the importance of strengthening oral health training and improving communication between pharmacists and dentists. A collaboration between pharmacists and dentists could improve patient care, according to 298 students.

**Conclusion:**

The results showed that, although students reported some familiarity with oral health conditions, their perceived knowledge remained limited. Since the study assessed perceived knowledge rather than objectively measured competence, the findings should be interpreted with caution. These findings suggest that strengthening oral health education in pharmacy studies could help improve pharmacists’ confidence in managing oral-health-related situations and appropriately referring patients when necessary.

In a world where more than 3.5 billion people are affected by oral diseases, it’s clear that they remain a global social issue.^40^ Although most of them are not serious in nature, they can nonetheless have an impact on people’s overall health, quality of life and well-being. Poor oral health can also have a substantial economic impact on populations and healthcare systems.^15,26,41
^


Oral health is largely dependent on modifiable socio-behavioural and environmental factors.^2^ Some factors, such as increasingly sugary and industrialised diets, smoking and poor oral hygiene habits, can have considerable consequences and explain the rising prevalence of oral pathologies worldwide.^27^


According to the WHO Global Oral Health Status Report (2022), around 29% of the world’s population has untreated caries on permanent teeth, making it the most widespread oral disease.^40^ Severe periodontitis affects 12.5% of the world’s population and ranks sixth among the most frequent chronic pathologies. Its repercussions on nutrition, aesthetics, and even social interactions make this pathology a major public health issue.^25,36
^ However, these conditions affecting the oral sphere are largely preventable, particularly through the implementation of preventive strategies targeting modifiable risk factors.^38^


At the national level, the situation is equally alarming. A report by the National Institute for Health Education and Prevention (INPES) and the French Union for Oral Health (UFSBD) indicates that between 33% and 50% of adults have at least one untreated decayed tooth requiring treatment.^24^ Periodontal diseases affect half of adults over the age of 35, and in 10% of cases, it involves a severe form of periodontitis.^8^


In France, there is currently high demand for dental care, which is difficult to meet fully, especially in underserved areas (long waiting times, geographic isolation, etc.). According to data from November 2024, there are on average 70 dentists per 100,000 inhabitants in France, placing the country below the average of the 14 European Union member states, which stands at 76 dentists per 100,000 inhabitants.^10,13
^ This difficulty in accessing care in the most underserved areas may lead patients to turn first to their pharmacist in order to receive advice or more prompt and accessible care.^30^ Indeed, according to 2021 data, the average distance between French municipalities and the nearest pharmacy is 3.8 km.^11^ Patients may prefer local pharmacists for many reasons, such as extended opening hours that allow patients to visit at any time, established relationships with regular patients that make local pharmacists trusted healthcare providers, and time-saving walk-in services.^30^ As a result, community pharmacists may be consulted daily by patients presenting with complaints or conditions related to oral health.^5^


These requests to the pharmacy are diverse, ranging from simple advice on an oral hygiene accessory to the management of a dental emergency. Community pharmacies are confronted on a daily basis with patients suffering from oral cavity disorders, such as gingivitis and periodontitis, oral mucosal lesions, dentine hypersensitivity, xerostomia, dental abscesses or halitosis.^19,22,32
^ Faced with this, the pharmacist should be able to meet patients’ needs, within the limits of his or her field of competence. Although pharmacists are drug specialists, their missions have evolved over the last few decades. Today, they play a major role in the management and prevention of oral pathologies.^19,30
^ They should be able to dispense the most appropriate medication for each situation, and inform patients about the possible oral and dental side effects of their treatment.^16,19
^ They also have to be able to provide appropriate advice, particularly on the oral hygiene products they sell, which represent a significant part of the pharmacy’s business and can play a major role in the prevention of oral diseases.^12^


To achieve this, pharmacists are expected to acquire basic knowledge of oral health and hygiene during their university training, and be familiar with the most common oral diseases and the recommendations for use of products sold in the pharmacy. They must demonstrate sound judgement in determining when it is appropriate to refer a patient to a dentist.^26^


Nevertheless, a number of studies show that pharmacists’ knowledge of oral health is still inadequate and could be improved.^4,5,7,15
^ Although a few studies have investigated the knowledge and practices of pharmacy students in this field, it would appear that no studies have been carried out specifically in France.^16,26
^


In France, initial training to obtain the Doctor of Pharmacy degree lasts 6 years. While pharmacy studies lead to a career as a pharmacist, opportunities are much broader depending on the specialisation pursued during the programme. Positions are available in community pharmacy, in the pharmaceutical industry, in research or in hospitals. This study aimed to establish the current state of perceived knowledge in odontology among sixth-year community pharmacy French students. The objective was to assess the interest and needs of future pharmacists in training in odontology, and to promote interprofessional collaboration between pharmacists and dentists, in order to guarantee quality care and effective management of the population.

## Methods and Materials

### Study Design and Protocol

This study was conducted using an online questionnaire in French, edited through Google Forms©. The questionnaire was specifically designed for this exploratory study following a review of the literature on oral health knowledge among pharmacists and pharmacy students, including previously published studies and pharmacy theses.^3,16,17,22
^ The questions were reviewed by faculty members from the departments of pharmacy and dentistry to assess their clarity and relevance. Before distribution, the questionnaire was preliminarily tested on a small group of pharmacy students, resulting in minor revisions to the wording to improve clarity.

The questionnaire was divided into three parts. The first part asked participants about any dental education they had received during their pharmacy studies. The second focused on students’ perceived knowledge of oral pathologies. The questionnaire used simplified terminology for oral and periodontal conditions in order to remain accessible to non-dental respondents and to reflect conditions commonly encountered in community pharmacy practice. The final section included additional questions, particularly on the relationship between the pharmacist and the dental surgeon. The questionnaire consisted of 20 questions: 5 single-choice questions, 14 multiple-choice questions and 1 question requiring an editorial response. Response time was estimated at 5 minutes. The French and English versions of the questionnaire are available in Supplementary Files 1 and 2. Prior to its diffusion, a protocol for this study was written, along with an information note for the participants. The note described the purpose of the survey and how it would be carried out. It informed participants that answering the questionnaire automatically implied their agreement to the use of the data collected, and that the questionnaire had been designed to guarantee participants’ anonymity. This protocol was approved by the Ethics Committee of the University Hospital of Rennes.^16,24
^


### Population

The target population consisted of community pharmacy students in their final year (sixth-year) in all faculties of mainland France. Only students enrolled in a faculty of pharmacy who had given permission for the questionnaire to be distributed were able to take part in the survey. Sixth-year pharmacy students outside the community pharmacy pathway were excluded from the study.

### Questionnaire Distribution

The 24 faculties of pharmacy in mainland France were contacted by e-mail and telephone between October 2023 and February 2024. Twenty faculties gave their authorisation, and the questionnaire was broadcast through them from March 2024 to June 2024, to 1352 students. Two reminder e-mails containing the link to the questionnaire were sent out, respectively, 1 month and 2 months after the first e-mail. The questionnaire was completed anonymously. Each student was asked to answer only once.

### Data Analysis

Prior to data analysis, a check was made for possible duplicates. Thus, two responses were removed from the study. Data were analysed using GraphPad Prism software version 10.2.3 (GraphPad Software, San Diego, CA, United States). The qualitative variables were compared using the Chi-square test if the conditions were met; otherwise, Fisher’s exact test was used. Sensitivity analyses were performed by excluding respondents from Rennes faculty, due to its particularly high participation rate and involvement in the study dissemination, and by stratifying faculties according to participation rates (high-response ≥ 30% versus low-response < 30%). A *p* value < 0.05 was considered statistically significant.

## Results

### Distribution by Pharmacy Faculties

A total of 318 out of 1352 students (23,5%) answered the questionnaire. Of all the students surveyed, almost a third (31.1%) were studying at the faculties of Montpellier (n = 26; 8.2%), Lyon (n = 25; 7.9%), Rennes (n = 24; 7.5%) and Nantes (n = 24; 7.5%). The three faculties with the highest participation rates were Rennes (46.2%), Nantes (43.6%) and Caen (39.6%) (Table 1).

**Table 1 Table1:** Distribution of respondents according to French pharmacy faculties and faculty-specific participation rates (ranking based on faculty participation rate)

Rank	Pharmacy faculty	Number of 6th-year community pharmacy students	Participants n (%)	Participation rate by faculty (%)
1	Rennes	52	24 (7.5)	46.2
2	Nantes	55	24 (7.5)	43.6
3	Caen	48	19 (6.0)	39.6
4	Dijon	54	19 (6.0)	35.2
5	Rouen	49	17 (5.3)	34.7
6	Lyon	74	25 (7.9)	33.8
7	Besançon	46	14 (4.4)	30.4
8	Tours	53	15 (4.7)	28.3
9	Angers	46	13 (4.1)	28.3
10	Montpellier	96	26 (8.2)	27.1
11	Reims	53	13 (4.1)	24.5
12	Nancy	88	20 (6.3)	22.7
13	Amiens	52	11 (3.5)	21.2
14	Clermont-Ferrand	65	13 (4.1)	20.0
15	Toulouse	78	15(4.7)	19.2
16	Paris-Saclay	97	14 (4.4)	14.4
17	Marseille	105	15 (4.7)	14.3
18	Bordeaux	62	6 (1.9)	9.7
19	Lille	149	14 (4.4)	9.4
20	Limoges	30	1 (0.3)	3.3
**TOTAL**		**1352**	**318 (100)**	


### Teaching of Dentistry During Pharmacy Studies

Of all the students surveyed, 22.6% had not received any courses in odontology during their pharmacy studies. However, the majority (96.9%) felt that these could be useful for their future practice in community pharmacy.

Students who had received training in dentistry (n = 246; 77.4%) indicated that the topics most frequently covered in their courses were oral hygiene (86.0%), periodontal diseases (68.3%), prevention of carious lesions (61.8%) and dental pain (46.3%). On the other hand, dental emergencies and dental trauma were among the least studied subjects (7.7% and 10.6%, respectively) (Fig 1).

**Fig 1 Fig1:**
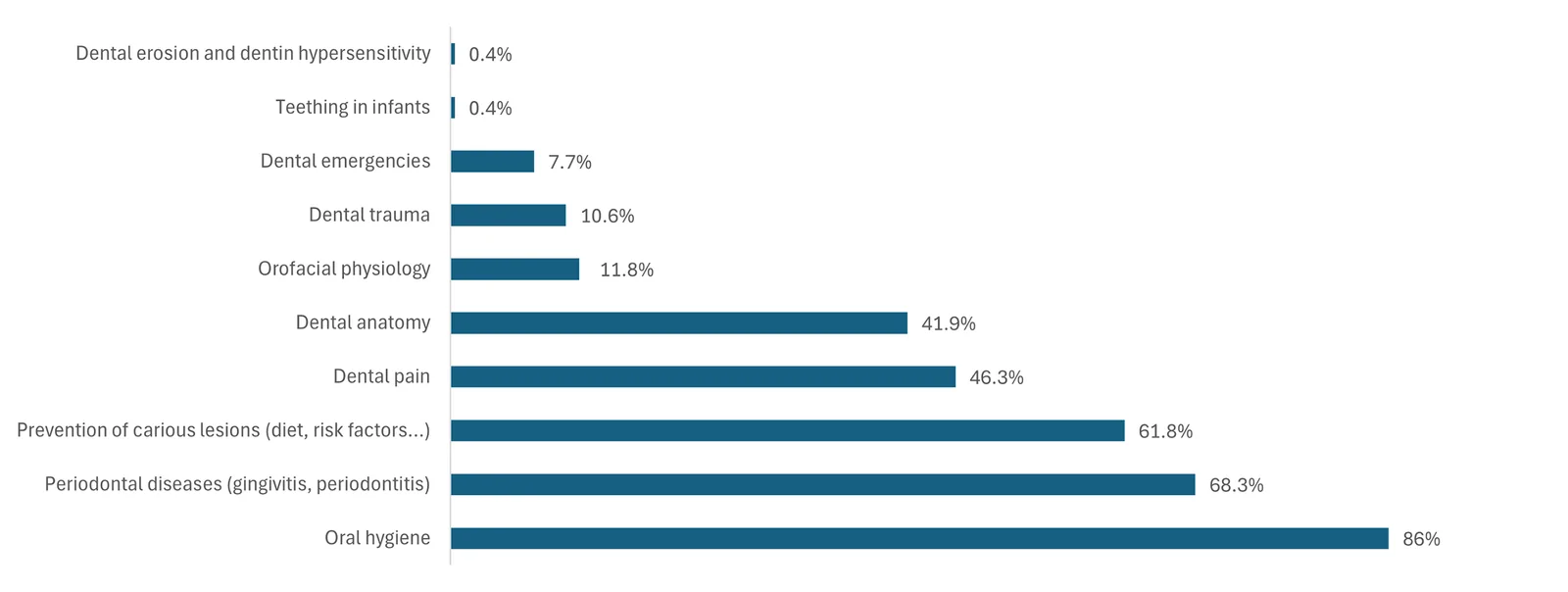
Topics covered during oral health training among the 246 students who reported having received dental education. Oral hygiene and periodontal diseases were the most frequently reported topics. Multiple answers possible.

Of these 246 students, the majority (72.0%) attended lectures given by pharmacy teachers, compared with only 24.4% by odontology teachers. Seventy-two students (29.3%) received training from pharmaceutical laboratory medical representatives, and for 25.0% of them, this was the only odontology training they received during their studies. Some students self-taught via professional journals or scientific papers (n = 8). 8 students received teaching from an external speaker (physician, dental surgeon, pharmacist), mainly in the form of case studies and clinical situations (Fig 2).

**Fig 2 Fig2:**
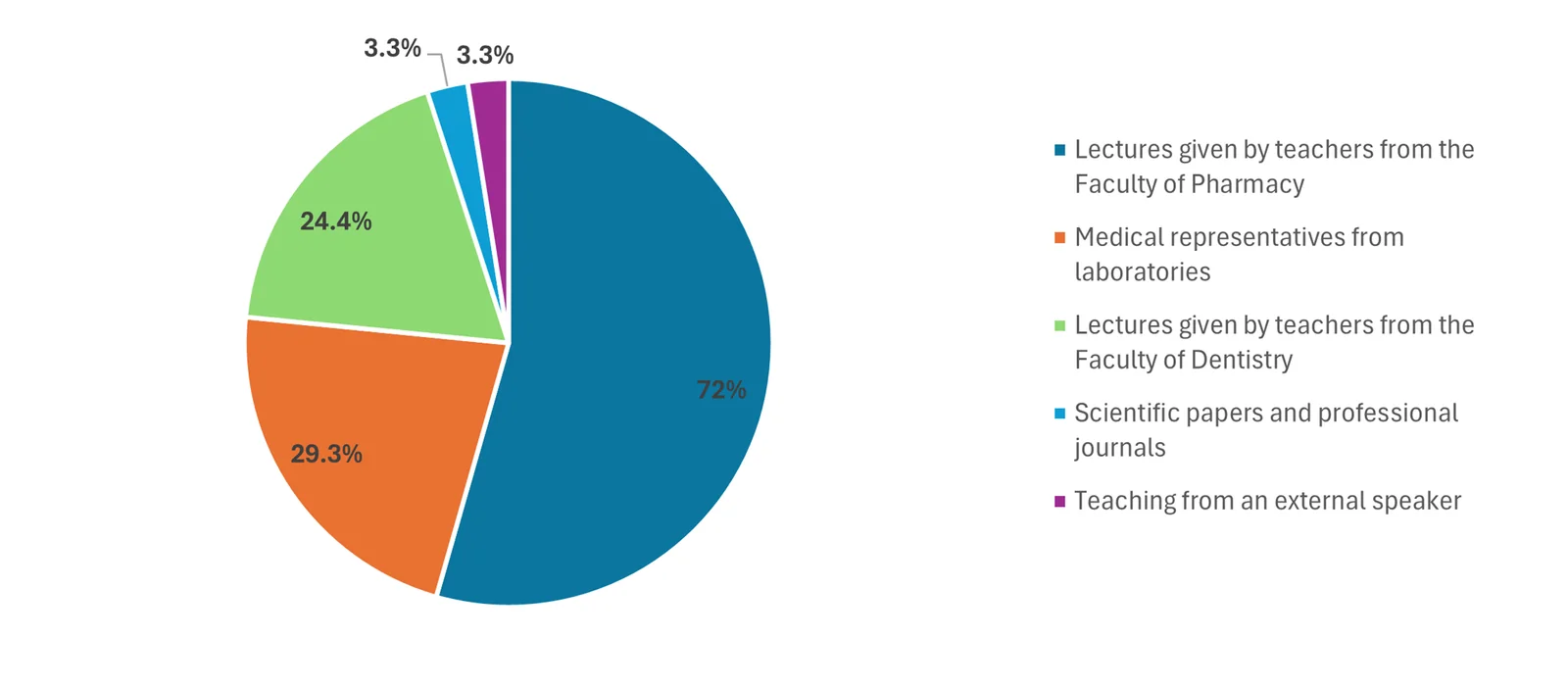
Type of dental training received by the 246 students who reported having received dental education. Most of the courses were taught by teachers from the faculty of pharmacy. Multiple answers possible.

Lastly, some students stated that they only had one or two hours of lectures or tutorials devoted to this type of teaching.

### Perceived Knowledge of Oral and Dental Diseases

Students were first asked to rate their perceived knowledge of oral pathologies (Table 2). Of the 318 respondents, 69.2% self-assessed their knowledge as ‘insufficient’, 28.3% as ‘good’ and 2.5% as ‘non-existent’. None self-assessed their knowledge as ‘excellent’. The majority of students who had received courses in dentistry considered their perceived knowledge to be ‘non-existent or insufficient’ (n = 165; 67.1%). It was also found that the proportion of students with ‘good’ perceived knowledge was significantly higher among students who had received courses in dentistry than among those who had not (*p* = 0.0007; Table 3). Sensitivity analyses were performed to assess the robustness of these findings. Analyses excluding respondents from Rennes faculty yielded results consistent with the primary analysis. Similarly, stratified analyses according to faculty participation rates showed comparable trends in both high-response (≥ 30%) and low-response (< 30%) faculties. Although the association between dental training and self-perceived knowledge did not remain statistically significant in the low-response subgroup, similar trends were observed (Suppl. File 3).

**Table 2 Table2:** Students’ perceived knowledge of oral and dental diseases. Results by number and percentage of total respondents (n = 318) (*multiple answers possible)

Perceived knowledge	Population n (%)
**How would you rate your perceived knowledge of oral pathologies? (n = 318)** Excellent Good Insufficient Non-existent	0 (0.0) 90 (28.3) 220 (69.2) 8 (2.5)
**Select the oral pathologies you have already heard about* (n = 318)** Mouth ulcer Herpes labialis Carious lesions Gingivitis Periodontitis Abscess Dentin hypersensitivity Pulpitis Halitosis Dental trauma Necrotizing periodontal diseases Dental cellulitis Alveolitis Pericoronitis	306 (98.7) 304 (95.6) 296 (93.1) 295 (92.8) 269 (84.6) 268 (84.3) 226 (71.1) 224 (70.4) 221 (69.5) 115 (36.2) 63 (19.8) 47 (14.8) 30 (9.4) 6 (1.9)
**Respondents who placed each condition among the five least-familiar conditions * (n = 318)** Pericoronitis Dental cellulitis Necrotising periodontal diseases Dental trauma Pulpitis Periodontitis Halitosis Dentin hypersensitivity Abscess Carious lesions Gingivitis Herpes labialis Mouth ulcer	301 (94.7) 277 (87.1) 275 (86.7) 185 (58.2) 169 (53.1) 68 (21.4) 61 (19.2) 55 (17.3) 33 (10.4) 17 (5.3) 13 (4.1) 9 (2.8) 9 (2.8)
**Do you know which prescription to use for the different pathologies mentioned above? (n = 318)** Yes, for all pathologies Yes, for certain pathologies No for all pathologies	2 (0.63) 278 (87.4) 38 (11.9)


**Table 3 Table3:** Association between oral health training and students’ perceived knowledge of oral pathologies. The proportion of students with ‘good’ perceived knowledge was significantly higher among students who had received courses in dentistry than among those who had not **p* < 0.05

		Perceived knowledge of oral pathologies		p value
	Total (n = 318)	Insufficient or non-existent (n = 228)	Good (n = 90)	0.0007*
Attended courses in dentistry				
Yes No	246 72	165 (67.1%) 63 (87.5%)	81 (32.9%) 9 (12.5%)	


Concerning oral pathologies that students have already heard about, the majority of them answered mouth ulcer (98.7%), herpes labialis (95.6%), carious lesions (93.1%), gingivitis (92.8%) and periodontitis (84.6%). More than three-quarters of students (78.6%; n = 250) selected between 6 and 10 pathologies. On average, 8 to 9 (8.4 ± 2.1) of the 14 proposed pathologies were selected. Conversely, the five least-known oral pathologies were pericoronitis (94.7%), dental cellulitis (87.1%), necrotising periodontal diseases (86.7%), followed by dental trauma (58.2%) and pulpitis (53.1%).

Only 2 of the 318 students questioned knew which prescriptions to use for which conditions. Thirty-eight participants (11.9%) did not know which prescription to use for any of the oral pathologies mentioned. However, there were no differences between participants who had received dentistry courses and those who had not (*p* = 0.16; data not shown).

### Management of Oral Health in Community Pharmacy

During their experience in pharmacy, almost all students (99.1%; n = 315) had already been confronted with patients presenting oral complaints or pathologies, at least twice a week for the majority (79.3%) (Table 4). The various complaints or pathologies rated as ‘most frequently encountered’ by these 315 students were herpes labialis (49.8%), mouth ulcers (44.1%), advice on oral hygiene products (43.5%) and dental pain (34.9%). Those mainly classified as ‘never encountered’ were dental cellulitis (77.8%), dental trauma (68.3%) and abscess (27.3%), although the latter had already been encountered by the majority of students.

**Table 4 Table4:** Students’ perceptions regarding the role of community pharmacists in oral health care. Most respondents considered that pharmacists have a role to play in oral health prevention and patient counselling. Results by number and percentage of total respondents (*multiple answers possible)

Oral health in community pharmacy	Population n (%)
During your internship in a pharmacy, have you ever been confronted with patients presenting oral complaints or pathologies? (n = 318) Yes No	315 (99.1) 3 (0.9)
**If yes, how often were you confronted with these patients over a 1-week period? (n = 315)** 0–1 time > 1 time 2–5 times 5–10 times > 10 times	65 (20.7) 249 (79.3) 192 (61.1) 40 (12.7) 17 (5.4)
**If yes, which complaints/pathologies were involved? * (n = 315)** **Most frequently encountered:** Herpes labialis Mouth ulcer Advice on oral hygiene products Dental pain Never encountered: Dental cellulitis Dental trauma Abscess	157 (49.8) 139 (44.1) 137 (43.5) 110 (34.9) 245 (77.8) 215 (68.3) 86 (27.3)
**Generally speaking, do you feel comfortable dealing with a patient presenting an oral complaint or pathology? (n = 318)** Yes No	111 (34.9) 207 (65.1)
**If not, why? * (n = 207)** Few or no university training in dentistry Diagnostic uncertainty Few or no communications with nearby dentists Few situations encountered in pharmacies	161 (77.8) 156 (75.4) 103 (49.8) 24 (11.6)
**Do you feel qualified to give advice on oral hygiene? (n = 318)** Yes, very comfortable Yes, but only for certain brands/products No	40 (12.6) 255 (80.2) 23 (7.2)
**Do you think you have a role to play in your patients’ oral health? (n = 318)** Yes No I don’t know	306 (96.2) 2 (0.6) 10 (3.1)


Generally speaking, more than half the students (n = 207) said they felt uncomfortable facing a patient presenting to the pharmacy with an oral complaint or pathology. The main reasons given were the absence or lack of university training in dentistry (77.8%) and diagnostic uncertainty (75.4%). For half of the respondents (49.8%), insufficient or no communication with the dentist practising nearby was also a reason. Other elements were mentioned, such as the apprehension of not detecting a situation that could have serious immediate consequences for the patient, or of dispensing a product that could worsen the patient’s condition. Students also stressed the fact that they did not always have a pharmaceutical solution to offer patients, and that they felt powerless in areas where there were too few dentists, with excessively long waiting times for an emergency consultation.

It was found that the proportion of students who felt confident in dealing with a patient presenting to the pharmacy for an oral health reason was significantly higher among students with ‘good’ perceived knowledge than among those with perceived knowledge classified as ‘insufficient or non-existent’ (*p* < 0.0001; Table 5). Additional sensitivity analyses excluding respondents from Rennes faculty showed findings consistent with the primary analyses. Stratified analyses according to faculty participation rates also yielded similar results, with the association between perceived knowledge and students’ comfort remaining statistically significant in both high-response and low-response faculty groups (Suppl. File 3). The majority of students (80.2%) said they felt comfortable providing oral hygiene advice, but only for certain brands or products. Only 12.6% were very comfortable and 7.2% not at all. No differences were found between students who had received dentistry courses and those who had not (*p* = 0.2; data not shown).

**Table 5 Table5:** Association between students’ perceived knowledge of oral pathologies and their comfort in managing oral health-related situations in community pharmacy practice. Higher self-perceived knowledge was associated with greater reported comfort. * *p* < 0.05.

		At ease with patients		p value
	Total (n = 318)	Yes (n = 111)	No (n = 207)	< 0.0001 *
Perceived knowledge of oral pathologies				
Insufficient or non-existent good	228 90	59 (25.9%) 52 (57.8%)	169 (74.1%) 38 (42.2%)	


Finally, of the 318 respondents, 306 (96.2%) felt they had a role to play in their patients’ oral health (Table 4).

### The Pharmacist-Dentist Relationship

According to their current perceived knowledge, 20.4% (n = 65) of the students surveyed felt that the prescriptions of their local dentist rarely corresponded to current recommendations (Fig 3).

**Fig 3 Fig3:**
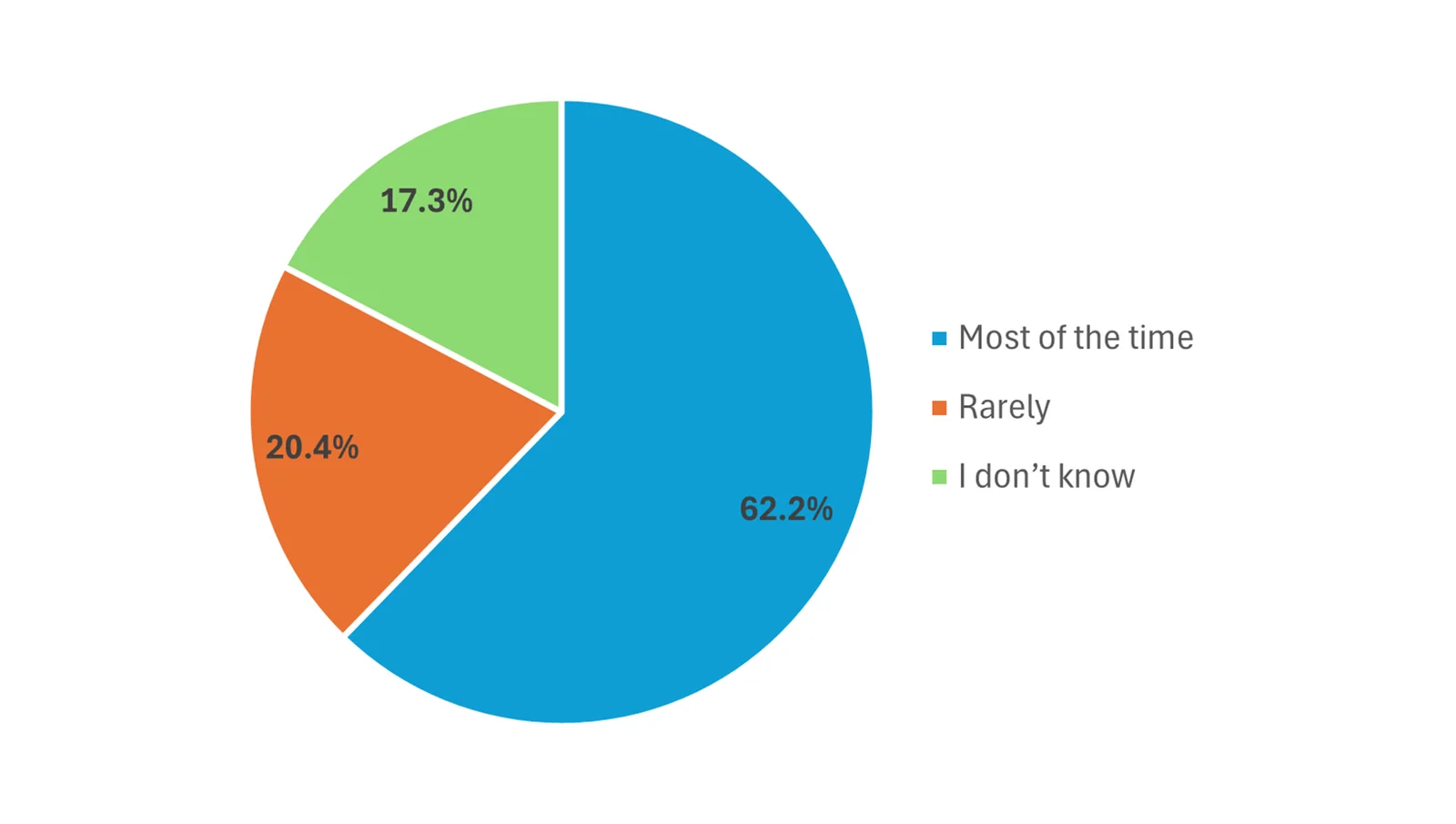
Students’ perceptions of the adequacy of local dentists’ prescriptions in relation to current recommendations (n = 318). Most respondents perceived these prescriptions as generally corresponding to current recommendations.

Of the 318 respondents, the majority (n = 298; 93.4%) felt that a collaboration between pharmacists and dentists could improve patient care (Table 6). Some students have suggested that collaboration should begin during their training at the faculty. Seventeen respondents mentioned the need to improve initial odontology training for future pharmacists, and 15 students drew attention to the fact that knowledge-sharing between students from different medical professions (such as workshops with role-plays, cross-internships, etc.) could greatly enhance their knowledge and cooperation.

**Table 6 Table6:** Students’ perceptions regarding pharmacist-dentist collaboration in oral health care. Most respondents considered collaboration between pharmacists and dentists to be important for patient care. Results by number and percentage of total respondents (*multiple answers possible)

The pharmacist-dentist relationship	Population n (%)
**Do you think that a collaboration between pharmacists and dentists could improve patient care? (n = 318)** Yes No/I don’t know	298 (93.4) 20 (6.6)
**If yes, how could it be set up or improved? *(n = 298)** Communication between healthcare professionals Meeting between healthcare professionals Creation of group exercise structures Improving initial training for pharmacy students Training between students of each discipline Training of pharmacists in dentistry	57 (19.1) 43 (14.4) 20 (6.7) 17 (5.7) 15 (5.0) 15 (5.0)
**Have you ever heard of the on-call system for dentists on Sundays and public holidays? (n = 318)** Yes No	184 (57.9) 134 (42.1)


Importantly, nearly half of the students (n = 134; 42.1%) were not aware of the on-call system for dentists on Sundays and public holidays. Respondents (n = 57; 19.1%) stressed the importance of effective communication between healthcare professionals to improve patient care. They would like to be able to contact the dentist easily and securely, via a secure messaging system, for example, particularly when the pharmacist requires a confirmation from the dentist (e.g. dosages, stock-outs). Several of them (n = 43; 14.4%) mentioned the idea of setting up inter-professional meetings on a territorial scale (communes/towns/departments) with the aim of exchanging practices and pooling knowledge.

## Discussion

To our knowledge, this is the first study carried out in France on pharmacy students’ perceived knowledge of odontology. The aim was to assess the interest and need for training in odontology among future pharmacists, and to promote interprofessional collaboration between pharmacists and dentists. The results showed that pharmacy students’ perceived knowledge and training in oral health do exist, although these remained insufficient to respond confidently to patients’ oral health requests and complaints. Most of them felt that real collaboration between the two professions, particularly through improved communication, would improve patient care.

Participation rates varied from faculty to faculty, ranging from 3.3% in Limoges to 46.2% in Rennes, suggesting non-response bias. This high level of participation could be explained by the involvement of the local student network and by the particular interest shown by Rennes pharmacy teachers in this survey, given that it was carried out in collaboration with some members of the teaching team.

A total of 22.6% of students surveyed did not benefit from any dentistry courses during their initial pharmacy training. However, it is possible that some students did not attend these courses despite the fact that they did take place (attendance not compulsory, courses available online, etc.). In a similar study carried out in California, 40.3% of students reported that oral health was not taught in any course of their pharmacy curriculum.^16^ In both studies, participants felt that training on oral health topics was insufficient.

With regard to teaching, we note that most lectures were delivered by pharmacy lecturers rather than odontology lecturers. Although this may be due to a number of factors (e.g. lack of available teaching slots or teaching staff), it undoubtedly shows that the pooling of knowledge between faculties of dentistry and pharmacy is not yet in place at all universities.

When we look at the topics covered in dentistry courses, they mainly concern the oral hygiene equipment sold in pharmacies, and the pathologies that can be prevented or treated by these products (in particular periodontal disease and carious lesions). These responses are not surprising, given that almost a third of students who have taken courses in dentistry have received training from laboratory medical representatives, who promote the same products to dispensing pharmacists. Although these interventions are useful for the future pharmacist’s practice, they appear insufficient. Indeed, many oral and dental conditions encountered in pharmacies and dental practices are not covered, as they do not fall within the field of expertise of medical representatives. It would therefore be interesting to extend this training in dentistry with courses given by teachers of odontology or private dental surgeons, who are experts in their field.

The findings concerning perceived knowledge of oral pathologies highlighted two major issues. Firstly, it appears that even though the students received training in dentistry, the majority considered their knowledge to be ‘non-existent or insufficient’. However, the results showed that there were significantly more students with ‘good’ perceived knowledge among those who had received courses in odontology compared with those who had not. These findings suggest that such courses may contribute to improving students’ perceived knowledge. Furthermore, there was a real demand from respondents, 96.9% of whom felt that training in dentistry would be useful for their future practice. The same trend was observed in the Gavaza et al. study carried out in California in 2015, where the majority of students felt they needed more training in this field.^16^ Secondly, several of the oral conditions least familiar to students corresponded to acute oral or dental situations potentially requiring rapid orientation or referral in community pharmacy practice, a topic taught to only 7.7% of them. Yet it is highly likely that these conditions require pharmacist advice, either before or after an appointment with the dentist. Training in dental emergencies could prove useful for better patient care. It should nevertheless be noted that the questionnaire explored periodontal diseases using broad terminology (e.g. periodontitis, necrotising periodontal diseases) rather than the complete 2017 World Workshop classification of periodontal and peri-implant diseases and conditions.^35^ The objective was not to assess detailed periodontal diagnostic competence, but rather students’ familiarity with oral conditions potentially encountered in community pharmacy practice. Consequently, the present study does not allow a detailed evaluation of pharmacy students’ knowledge of specific periodontal or peri-implant entities.

It should also be emphasised that the questionnaire assessed self-perceived knowledge and familiarity with oral health terminology rather than objectively measured clinical competence. No clinical vignettes or validated knowledge-assessment tools were included. Consequently, the findings should be interpreted as reflecting students’ perceptions and awareness of oral conditions rather than demonstrated diagnostic or therapeutic abilities. In addition, although the questionnaire was reviewed by faculty members from the departments of pharmacy and dentistry and pre-tested among pharmacy students, no formal psychometric validation or reliability assessment was conducted.

This survey showed that oral health care in French pharmacies is considerable: almost all students (99.1%) have already seen a patient for an oral complaint or pathology, compared with only 32.0% of students of a Turkish university, according to the study by Ozler et al.^26^ This discrepancy can, however, be explained by different inclusion criteria: in the Turkish study, all students from Hacettepe University in Ankara (all years and all specialities) were included, whereas in this study, only sixth-year community pharmacy students from 20 French faculties were included. Moreover, the majority of participants in the present survey (around 80%) stated that they were consulted at least twice a week on oral health issues. According to a French pharmacy thesis conducted in 2020, this frequency is in the order of 10 to 30 times a week in community pharmacies.^22^


It seems that the oral pathologies most familiar to students were among those classified as ‘frequently encountered’ in the pharmacy: mouth ulcers and herpes labialis. This is not surprising, since these are two frequent dermatological conditions, the symptomatic treatment of which can be dispensed by pharmacists without prescription.^18,20,33
^ Carious lesions and periodontal disease were also well known to these students. Apart from the fact that these are very common oral pathologies, they can also be prevented and treated, among other things, by oral hygiene products sold in pharmacies.^6^ In addition, dispensing advice for these products is a situation frequently encountered by participants. Although students appeared to be relatively familiar with oral hygiene products and related advice, it should be pointed out that some of them felt comfortable only with certain brands or products, which could be related to the type of training they received or the training provider (teacher or laboratory representative).

As for the lesser-known oral and dental conditions, two of them were classified by the majority as ‘never encountered’ at the pharmacy: dental cellulitis and dental trauma. Nevertheless, it’s worth noting that a sizeable number of students have already experienced them (22.2% and 31.7%, respectively). Their early management by pharmacists could have a favourable impact, not only on the prognosis of the tooth, but also on the patient’s general state of health. That’s why pharmacists should have a basic understanding of dental emergencies, be able to identify them and know what to do at their level, delivering the right advice and medicines for the situation, and making the most appropriate referrals if necessary. In this context, the competencies expected from future community pharmacists in oral health should be more clearly defined. Beyond a basic understanding of oral diseases, pharmacists should ideally be able to recognise common oral conditions, identify warning signs requiring urgent dental referral, provide appropriate advice on oral hygiene products, detect oral adverse drug reactions, manage minor oral complaints within their professional scope, and refer patients to appropriate dental care when necessary. Strengthening these competencies through interprofessional education could improve both pharmacists’ confidence and the quality of patient care. These competencies are consistent with current recommendations promoting interprofessional collaboration between pharmacists and dental professionals.^21,30
^ In France, some interprofessional practice-support initiatives and guidance documents developed jointly by pharmacists’ and dentists’ professional organisations already encourage community pharmacists to improve their ability to identify oral emergencies, provide first-line advice, and appropriately refer patients.^1,37
^ In addition, almost all respondents (96.2%) felt they had a role to play in their patients’ oral health, a trend found in most studies on this subject.^5,7,9,15–17,19,23,26,29,31^


The results also showed that those with ‘good’ perceived knowledge were significantly more likely to feel at ease in front of a patient than those with ‘insufficient or non-existent’ perceived knowledge. These findings suggest that improving oral health education, in particular during initial training, could help students feel more confident when dealing with oral health issues. According to a study carried out by Al-Saleh et al in Saudi Arabia, only 26.5% of the community pharmacists were confident in providing oral health advice.^3^ It is, however, important to mention that the oral cavity presents specific challenges. It is a confined and poorly lit space that requires a detailed anamnesis, often supplemented by imaging and clinical tests to establish a diagnosis. Therefore, the uncertainty experienced by pharmacists may stem not only from insufficient knowledge but also from the intrinsic diagnostic complexity of oral conditions.

This underscores the importance of pharmacists and dentists complementing each other in patient care. Ideally, their partnership should begin with initial training, notably through interprofessional courses. Experiments conducted on this subject, such as that by Pogge et al. in America in 2016, showed that students following the interprofessional education programme improved their skills and knowledge, compared with non-participants.^28^ This type of experience is positively perceived by pharmacy and odontology students.^14,34,39
^ It raises their awareness of the role of each profession and of collaborative practices, helping them to prepare for their future profession through a more global vision.^21^


When pharmacy students were asked how collaboration with dentists could be established or improved, some emphasised the importance of reciprocal knowledge-sharing during their studies, in the form of role-plays and workshops. Other students attached particular importance to improving the initial training of both paths, in order to consolidate their knowledge in one or other of the fields. In addition, it would seem appropriate for teachers of dentistry and/or liberal professionals to participate to a greater extent in the training of pharmacy students, in order to contribute their expertise and experience. The need for dental training among future pharmacists is becoming increasingly apparent. This problem seems to have persisted over the years, since according to a pharmacy thesis carried out in 2020, nearly 80% of French dispensing pharmacists felt that they had not acquired sufficient knowledge and training in the oral-dental field.^22^


Students also highlighted the lack of communication between healthcare professionals, which is essential for effective patient care. As the study by Bawazir et al showed, sometimes these healthcare professionals, working in close proximity to one another, have never met.^5^ To improve this collaboration, respondents suggested setting up interprofessional meetings to encourage continuing education in each speciality and harmonise knowledge.

Although this study highlighted limited perceived oral health knowledge among pharmacy students, future studies could also explore pharmacy-related knowledge and interprofessional perceptions among dental students. It would therefore be appropriate to conduct a similar study among the latter. In addition, the results revealed that 20.4% of students surveyed perceived discrepancies between dental prescriptions and current recommendations. A similar percentage was found in a pharmacy thesis conducted among French pharmacists.^12^ However, given that the majority of respondents also reported limited perceived knowledge in oral health, these findings should be interpreted cautiously and may reflect uncertainty regarding prescribing recommendations rather than actual inadequacies in dental prescribing practices.

If they are to work as a team, it seems obvious that the relationship between dentists and pharmacists needs to be established as early as their initial training, with joint teaching and mutual sharing of knowledge. For future pharmacists, these exchanges will undoubtedly enable them to be more confident when dealing with patients, particularly in dental emergencies. They will also be in a better position to provide them with appropriate advice and refer them to the right structure. Collaboration between healthcare providers from different professions, including nurses, physicians and other health professionals, can facilitate early detection of diseases and rapid referral of patients.^30^


## Conclusion

This study provided an overview of the perceived knowledge in odontology of sixth-year community pharmacy French students. Although they have acquired some basic knowledge during their studies, it appears to be insufficient. The need for training in dentistry among future pharmacists is becoming increasingly apparent. Based on this observation, it would be advisable to integrate more in-depth training in odontology into the curriculum of pharmacy students. Without adequate training programmes, the community pharmacists may not be able to provide appropriate advice to promote oral health or treat oral health problems.

## References

### Appendix

https://www.quintessence-publishing.com/quintessenz/journals/articles/downloads/ohpd_2026_8265_gentet_suppl_file_1.docx

https://www.quintessence-publishing.com/quintessenz/journals/articles/downloads/ohpd_2026_8265_gentet_suppl_file_2.docx

https://www.quintessence-publishing.com/quintessenz/journals/articles/downloads/ohpd_2026_8265_gentet_suppl_file_3.docx

## References

[ref2] Al Subait AA, Alousaimi M, Geeverghese A, Ali A, El Metwally A (2016). Oral health knowledge, attitude and behavior among students of age 10–18 years old attending Jenadriyah festival Riyadh; a cross-sectional study. Saudi J Dental Res.

[ref3] Al-Saleh H, Al-Houtan T, Al-Odaill K, Al-Mutairi B, Al-Muaybid M, Al-Falah T (2017). Role of community pharmacists in providing oral health advice in the Eastern province of Saudi Arabia. Saudi Dent J.

[ref4] Baseer MA, Mehkari MA, Al-Marek FAF, Bajahzar OA (2016). Oral health knowledge, attitude, and self-care practices among pharmacists in Riyadh, Riyadh Province, Saudi Arabia. J Int Soc Prev Community Dent.

[ref5] Bawazir OA (2014). Knowledge and attitudes of pharmacists regarding oral healthcare and oral hygiene products in Riyadh, Saudi Arabia. J Int Oral Health.

[ref6] Bernabe E, Marcenes W, Abdulkader RS, Abreu LG, Afzal S, Alhalaiqa FN (2025). Trends in the global, regional, and national burden of oral conditions from 1990 to 2021: a systematic analysis for the Global Burden of Disease Study 2021. Lancet.

[ref7] Blebil A, Dujaili J, Elkalmi R, Tan HLK, Tai MS, Khan TM (2020). Community pharmacist’s role in providing oral health-care services: findings from Malaysia. J Pharm Bioallied Sci.

[ref8] Bourgeois D, Bouchard P, Mattout C (2007). Epidemiology of periodontal status in dentate adults in France, 2002–2003. J Periodontal Res.

[ref9] Buxcey AJ, Morgaine KC, Meldrum AM, Cullinan MP (2012). An exploratory study of the acceptability of delivering oral health information in community pharmacies. N Z Dent J.

[ref14] Dresser J, Barazanchi A, Meldrum A, Marra C, Wilby KJ (2021). Identifying perceptions and themed learning outcomes between pharmacy and dentistry students through interprofessional education and collaboration in the dental clinic. Curr Pharm Teach Learn.

[ref16] Gavaza P, Ta N, Mosavin R (2016). An investigation of pharmacy students’ perceptions of their oral health knowledge and education: a preliminary study. Curr Pharm Teach Learn.

[ref17] Hajj A, Hallit S, Azzo C, Abdou F, Akel M, Sacre H (2019). Assessment of knowledge, attitude and practice among community pharmacists towards dental care: a national cross sectional survey. Saudi Pharm J.

[ref20] Jones A, Sturrock A, Elliott E, Gussy M, Maidment I, Nelson D (2024). Community pharmacists’ perceptions on managing people with oral health problems – a prioritisation survey. J Oral Rehabil.

[ref21] Lygre H, Kjome RLS, Choi H, Stewart AL (2017). Dental providers and pharmacists: a call for enhanced interprofessional collaboration. Int Dent J.

[ref24] Ménard C, Grizeau-Clemens D, Wemaere J (2016). Santé bucco-dentaire des adultes. Évolutions.

[ref25] Nascimento GG, Alves-Costa S, Romandini M (2024). Burden of severe periodontitis and edentulism in 2021, with projections up to 2050: the Global Burden of Disease 2021 study. J Periodontal Res.

[ref26] Ozler CO, Dalgara T, Sahne BS (2023). Oral care and maintenance habits among pharmacy students. Am J Pharm Educ.

[ref28] Pogge EK, Reynolds SC, Davis LE, Storjohann TD (2018). A pilot study on an interprofessional course involving pharmacy and dental students in a dental clinic. Am J Pharm Educ.

[ref29] Priya S, Madan Kumar PD, Ramachandran S (2008). Knowledge and attitudes of pharmacists regarding oral health care and oral hygiene products in Chennai city. Indian J Dent Res.

[ref30] Rajiah K, Lim WK, Madeline Teoh PL (2021). Community pharmacists’ knowledge, attitudes and practices towards oral healthcare and its management: a systematic review. Int J Clin Pract.

[ref32] Taing MW, Choong J, Suppiah V (2023). A cross-sectional survey exploring Australian pharmacists’ and students’ management of common oral mucosal diseases. Pharmacy (Basel).

[ref33] Taing MW, Ford PJ, Gartner CE, Freeman CR (2016). Describing the role of Australian community pharmacists in oral healthcare. Int J Pharm Pract.

[ref34] Theodorou J, Rotz M, Macphail L (2018). Designing and evaluating an interprofessional practice experience involving dental and pharmacy students. Am J Pharm Educ.

[ref35] Tonetti MS, Greenwell H, Kornman KS (2018). Staging and grading of periodontitis: framework and proposal of a new classification and case definition. J Clin Periodontol.

[ref36] Tonetti MS, Jepsen S, Jin L, Otomo-Corgel J (2017). Impact of the global burden of periodontal diseases on health, nutrition and wellbeing of mankind: a call for global action. J Clin Periodontol.

[ref38] Welti R, Jones B, Moynihan P, Silva M (2023). Evidence pertaining to modifiable risk factors for oral diseases: an umbrella review to inform oral health messages for Australia. Aust Dent J.

[ref39] Williamson K, Milone A, Reibel Y (2023). Evaluating an interprofessional pharmacy and dental hygiene case-based learning activity with student reflections. Curr Pharm Teach Learn.

